# Iatrogenic Bile Duct Injury Associated with Anomalies of the Right Hepatic Sectoral Ducts: A Misunderstood and Underappreciated Problem

**DOI:** 10.1155/2009/153269

**Published:** 2009-06-04

**Authors:** Nitin Babel, Sujit V. Sakpal, Prakash Paragi, Jason Wellen, Stephen Feldman, Ronald S. Chamberlain

**Affiliations:** Department of Surgery, Saint Barnabas Medical Center, Livingston, NJ 07039, USA

## Abstract

Although laparoscopic cholecystectomy (LC) has been widely accepted as the standard of care, it continues to have a higher complication rate than open cholecystectomy. Bile duct injury with LC has often been attributed to surgical inexperience, but it is also clear that aberrant bile ducts are present in a significant number of patients who sustain biliary injuries during these procedures. We present three cases of right sectoral hepatic duct injuries which occurred during LC and provide a discussion of the conditions which are likely to lead to these injuries, as part of a strategy to prevent them.

## 1. Introduction

**Figure 1:**
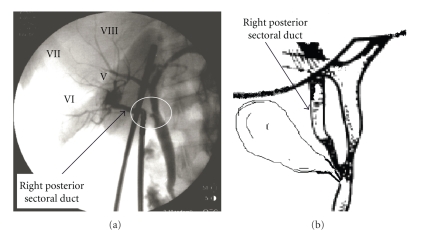
(a) An intraoperative cholangiogram (IOC) showing transection site (encircled) of the right posterior sectoral duct (drains segments VI and VII as indicated) in a 67-year-old female during routine laparoscopic cholecystectomy (*Strasberg* Type C injury). (b) Sketch illustration of the biliary anatomy as perceived from the cholangiogram.

**Figure 2:**
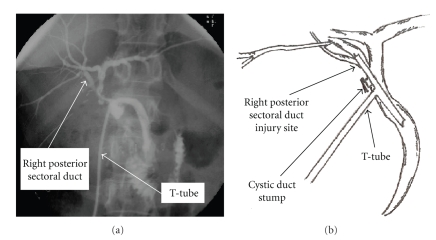
(a) Completion T-tube cholangiogram following a T-tube (14 Fr.) repair of right posterior sectoral duct injury in a 54-year-old female during laparoscopic cholecystectomy (*Strasberg* Type C injuriy). The study demonstrates absence of a leak/stricture postrepair. (b) Sketch illustration of the biliary anatomy as perceived from the cholangiogram.

**Figure 3:**
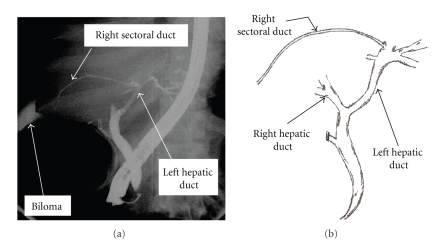
(a) An ERCP image shows fluid collection (biloma) adjacent to an injured right sectoral duct remnant with anomalous communication to the left biliary system in a 62-year-old female who presented 10 days after laparoscopic cholecystectomy with fever and jaundice. (b) Sketch illustration of the biliary anatomy as perceived from the cholangiogram.

Bile duct injuries remain one of the most devastating complications of both open and laparoscopic cholecystectomy (LC). Heuristic errors of perception in which surgeons fix anatomical reference points incorrectly and commence dissection in an area of danger is perhaps the most common cause of bile duct injuries. Injury prevention theories have suggested that strategies to prevent injuries would be most effective at the time of anatomical identification and orientation, and prior to dissection. We present three cases of right sectoral duct injuries, two of which had right posterior sectoral duct (RPSD) injuries noted on intraoperative cholangiogram (IOC) (*Strasberg* type C injuries) (Figures 1, 2), while the third had a right sectoral duct injury noted on postoperative endoscopic retrograde cholangiopancreatography (ERCP) (*Strasberg* type A injuries) (Figure 3) (Table 1). Biliary injuries were primarily repaired and stented over 14 French (Fr.) caliber T-tubes brought through separate choledochotomies in the first two cases, while an ERCP was utilized for postoperative evaluation and therapy in the third.

Although most leaks originate from the cystic duct (CD) stump and common bile duct (CBD), those from aberrant sectoral bile ducts are also common but rarely discussed. Biliary tree anomalies are present in up to 25% of patients, with aberrant right hepatic ducts being the most common [4]. Aberrant sectoral duct arise most commonly from the right liver and drain into the common hepatic duct (CHD) or CD (Figure 1(b)). An aberrant right sectoral hepatic duct usually represents the only route of biliary drainage for the portion of the right hepatic lobe it drains. The most dangerous sectoral variant is when the CD runs along side of a low-lying aberrant right sectoral duct. Most commonly this is the RPSD, which drains segments 6 and 7 and is present in 4.8–8.4% of the population [4, 5]. Injuries to these ducts are likely underreported since they may be asymptomatic and often unrecognized as the injured area atrophies over time.

## 2. Illustrative Cases

Three patients underwent LC and sustained iatrogenic RPSD injuries requiring operative or endoscopic management. 

In the first two cases, a review of IOCs (Figures 1, 2) demonstrated aberrant biliary anatomy and injuries to low-lying RPSDs. A tangential injury to the RPSD in both cases was successfully recognized and repaired primarily by duct-to-duct anastomosis. T-tube (14 Fr.) stenting of the repair was performed through a separate choledochotomy, with the upper arm of the T-tube crossing the injured RPSD. Post-repair cholangiogram obtained through the T-tube revealed opacification of all segments of the right liver without extravasation or stricture (Figure 2). The postoperative course of these two patients was uncomplicated. If the injuries had not been recognized intraoperatively, bile leaks would have occurred. These injuries may have escaped detection and opacification at ERCP due to the lack of communication of the injured ducts with main biliary channels, thereby rendering endoscopic management impossible. Such injuries should be suspected when a bile leak persists despite “normal” cholangiography and there is a presumed failure of a “CD stump leak” to close after biliary stent placement.

In the third case, an ERCP (Figure 3) revealed the bile leak to arise from an injured aberrant right sectoral duct with anomalous communication to the left biliary system. Successful therapy with ERCP, sphincterotomy and biliary stent placement was performed.

## 3. Discussion

Pilots and astronauts need to make accurate judgments of motion to perform critical aerospace tasks safely and effectively to avoid disaster. Similarly, surgeons must make perceptual judgments during surgery to avoid complications. Although laparoscopic biliary surgery is over 15 years old, biliary injuries continue to be a major source of morbidity and concern for surgeons. Despite continued efforts, the rate of injury has leveled off at 0.5% for LC. Typically, biliary injuries occur either due to technical problems or anatomical misidentification of the CD, latter being the most common cause. Two structures most often misidentified as the CD are the CBD or an aberrant right hepatic duct (RHD). The resultant injuries are of two main types—CBD mistaken for CD is clipped and divided; and the segment of an aberrant RHD (between entry of the CD and junction of the CHD) is misidentified and either ligated, transected or injured during dissection in Calot's triangle. 

Way et al. [3] analyzed 252 patients with laparoscopic bile duct injuries and concluded that these injuries stem principally from visual perceptual illusion, and not from errors of skill, knowledge, and judgment. This misperception theory is compelling in that even on identification of irregularities corrective feedback did not occur, thus supporting the notion that the native human mindset to hold firm to our assumptions is strong. In spite of excellent laparoscopic visualization, misidentification of extrahepatic accessory bile ducts during LC is a frequent cause of complications. IOC is good at avoiding misidentification of the CBD, but poor at detecting an aberrant RHD depending on the location of the CD puncture site. Nevertheless, performing complete circumferential dissection of the gallbladder neck and CD before cholangiography or clip placement should enable visualization of any variant anatomy. This recommendation was not followed in two of our cases, hence non-circumferential dissection and early clipping was performed. A choledochotomy for the cholangiogram was mistakenly made into an aberrant RHD instead of the CD (Figure 1). This type of injury has been described as a class IV “Lawrence Way” injury [3] due to misidentification of the RPSD as the CD resulting in a lateral injury, transection or ligation of an unseen low-lying RHD.

When correctly interpreted, routine IOC increases the chances of recognizing bile duct injuries, but it may not lower their incidence. This prompts us to conclude that more than a routine IOC may be essential to prevent biliary injuries. Magnetic resonance cholangiopancreatography (MRCP) is an excellent noninvasive imaging modality that is capable of demonstrating aberrant biliary anatomy [6]. Izuishi et al. [7] analyzed anatomical variations of the biliary tree with multislice-computed tomography (MCT) cholangiography in 113 patients. MCT cholangiography provided clear images of aberrant bile ducts in 18 patients—major type (draining liver segment) in 9 (8%) and minor type (draining liver subsegment) in 9 (8%). Alibrahim et al. [8] demonstrated the efficacy of spiral CT-iv cholangiography with 3D reconstructions for imaging the biliary tree. Using this technique excellent visualization of the biliary anatomy was achieved with good opacification of at least first-order or third-order bile ducts in 91% or 84% of patients, respectively. Despite their efficacy, routine ERCP, MRCP, and contrast-enhanced MCT cholangiography are expensive tools, and should be reserved for select cases. 

Irrespective of information derived on imaging, improvisation of surgical technique is necessary to avoid biliary injuries. Careful dissection, “conclusive identification” of anatomy with clearing of the hepatocystic triangle of all fat, and determination of “endpoint” in the dissection is pivotal in prevention of intraoperative complications. In 1995 Strasberg et al. [2] introduced a technique for “conclusive identification” of the cystic structures at LC based on a “critical view of safety”. Other important strategies include the use of a 30-degree telescope which provides *en face* view of the Calot's triangle and its intermittent withdrawal gives the surgeon an overall perspective and spatial orientation. Simulation-based surgical training and the availability of biologically-based models of human body architecture will permit virtual reality to serve as quantitative models of human self-motion perception. Performance analysis in heading estimation and related motion perception tasks will aid in the design of training regimes for surgeons in the near future. Robotic instruments to simulate the motions of the surgeon's wrist and the use of 3D imaging to immerse the surgeon in a 3D video operating field may also offer potential solutions to the inherent problems of traditional laparoscopic surgery. Though exciting, these technologies are still in an early stage of development, and each device entails its own set of challenges and limitations. 

Despite all the emerging advances bile duct injuries will still occur, and if they do conversion to open procedure is usually indicated. Repair should be attempted by general surgeons only if the techniques of dissection and reconstruction are common and familiar, and every effort should be made to conserve bile duct length. Repair techniques for minor injuries to the lateral wall of the CHD or CBD are familiar to most general surgeons, but injuries to aberrant ducts and those at or above the confluence of hepatic ducts require operative skills more likely to be available at specialized hepatobiliary units. When such expertise is unavailable at the time of injury, closed suction drains should be placed in the region of hepatoduodenal ligament and referral made expeditiously.

## Figures and Tables

**Table 1:** Classification of bile duct injuries based on either bile leaks or location of strictures. Right hepatic duct (RHD), common bile duct (CBD), common hepatic duct (CHD). The classification of injuries identified in our patients is highlighted.

Classification/Year		Bismuth [1](1982)	Strasberg [2](1995)	Way [3](2003)
Bile Leak	Cystic duct or terminal biliary radical leak		A	
	From CBD/CHD, no tissue loss		D	I
	From CBD/CHD, tissue loss			II
	From RHD (posterior sectoral)		C	**IV**
	CBD/CHD transection/occlusion			III
Strictures	CBD stricture			
	CHD >2 cm	I	E1	III
	CHD <2 cm	II	E2	III
	Hilar stricture, intact confluence	III	E3	III
	Hilar stricture, disrupted	IV	E4	III
	confluence			
	Obstructed R posterior hepatic	**V**	**B**/**E**5	IV
	Duct +/− CBD/CHD stricture			
